# Sensory driven neurophysiological mechanisms of concussion: a parsimonious and falsifiable theory

**DOI:** 10.3389/fneur.2025.1547786

**Published:** 2025-04-30

**Authors:** Donald Krieger, Paul Shepard, Anthony Kontos, Michael W. Collins, Ava Puccio, Shawn R. Eagle, Walter Schneider, David O. Okonkwo

**Affiliations:** ^1^Department of Neurological Surgery, University of Pittsburgh, Pittsburgh, PA, United States; ^2^Department of Physics and Astronomy, University of Pittsburgh, PA, United States; ^3^Department of Sports Medicine, University of Pittsburgh, Pittsburgh, PA, United States; ^4^Department of Psychology, University of Pittsburgh, Pittsburgh, PA, United States

**Keywords:** head trauma, traumatic brain injury, concussion, neurophysiology, CamCAN, magnetoencephalagraphy

## Abstract

Every time a person sustains a blow to the head, they receive multiple atypical sensory inputs, often including pain. These directly stimulate the central nervous system. Yet, sensory input as a causal agent of neurophysiological dysfunction and post-concussion symptoms has never been explored. A new theory is proposed of sensory driven neurophysiological mechanisms of concussion (i) which are causally linked to the momentary blow to the head, (ii) whose time courses and other properties correspond to those observed to date for acute, sub-acute, and chronic symptoms, and (iii) which give rise to testable questions with experimentally measurable consequences. The primary assertion of the theory is that trauma induced excitation of key brain regions including the salience network (SN) and locus coeruleus (LC) can produce persistent dysfunctional alterations in the stable patterns of network excitability on which symptom-free neurological function depends. This mechanism is in play with any physical trauma, with or without a blow to the head. That is because atypical, painful, and otherwise high intensity sensory stimuli excite the SN and thence the LC, inducing plasticity widely in the brain. Many of those sensory stimuli may persist through the recovery period and while the brain is plastic, enable one or another network to learn altered and potentially dysfunctional patterns of network excitability. The secondary assertion of the theory is that with a blow to the head, convergence of high-intensity sensory stimuli within the brainstem and midbrain can cause neurophysiological coupling between brainstem nuclei which normally function independently, i.e. brainstem crosstalk (BCT). It is BCT which causes the signs and symptoms specific to head trauma, e.g., loss of consciousness, and oculomotor and vestibular dysfunction. The theory’s reliance on sensory input emphasizes the importance of putative mechanisms whose initiating cause is known to have been present for every head trauma. This is in contrast to the century-long focus on mechanisms whose initiating cause, brain injury, is undetectable by clinical exam, neuroimaging, and bioassay in fully 60% of all head trauma, i.e., 70–75% of all mild TBI. As formulated and described, the theory is readily testable and falsifiable.

## Introduction: motivation for the theory

In this paper, we use the term “concussion” to mean a blow to the head which results in what is defined in clinical practice and in the literature as mild TBI, i.e., Glasgow Coma Score (GCS) = 13–15 and rapid onset of neurological impairment which typically resolves spontaneously and quickly (1, 2), i.e., “concussion” and “mild TBI” as used in this paper are synonymous. Though both terms refer to clinical incidents with and without documented brain injuries, concussion does not imply by its name that brain injury occurred. We emphasize this because a key purpose of the theory is to account for the symptoms of concussion without assuming brain injury as a cause. Our presumption is that even for patients in whom a brain injury occurred, sensory driven mechanisms are also in play and may contribute to or even outstrip the effects of injury-driven mechanisms to cause symptoms.

The global yearly incidence of TBI of all severities is about 69 million, i.e., 939 cases per 100,000 people or just under 1% (3). Mild TBI, i.e., GCS 13–15 (1), affects about 55.9 million, i.e., 81% of the total. In 2017, the incidence of mild TBI in the United States was about 3.8 million with 53% reporting persistent symptoms and functional impairment a year after their concussion (4).

As detailed in the review of theories of concussion in Supplementary material C, our current understanding of the signs and symptoms of concussion relies on two assumptions: (i) the signs and symptoms are due to brain injury and (ii) the patient’s pathology was caused by the mechanical impact of the concussion. The assumption of brain injury provides a putative mechanism by which the momentary time course of the concussion can produce symptoms with a time course extending indefinitely. However, these assumptions disregard the following:

(1) 70–75% of all mild TBI cases show no evidence of structural or other internal injury on clinical exam, neuroimaging (5), or biochemical testing (6–8). Surely there is a real question that brain injury exists in head trauma cases where no objective evidence can be found. Surely any understanding of neuropathology, symptoms, and treatment based on the unsupported assumption of brain injury is equivocal. And surely any research effort aimed to assess the efficacy of a treatment or the sensitivity and specificity of a biomarker would be significantly weakened if applied to a mixed group of those with and without brain injury.
(2) In all mild TBI cases, the central nervous system is directly excited by atypical, high intensity, and often painful sensory input. Though not capable of producing a brain injury of any kind, this direct neurophysiological excitation of the brain might, in some patients, produce signs and symptoms by causing disruptions in the neurophysiological mechanisms on which normal brain function depends. Surely it makes sense to identify and test experimental hypotheses founded on the possibility that sensory-driven neurophysiological mechanisms are at least partly responsible for the signs and symptoms of concussion.

Our understanding of the neurophysiological mechanisms which underlie most of the symptoms is minimal. Here are two examples:

(1) While it makes intuitive sense that a skull fracture, a white matter injury, or a focal or diffuse brain injury disclosed by blood on a computed tomography (CT) scan or signal changes on magnetic resonance imaging (MRI) would cause symptoms, no widely accepted mechanistic understanding of the causal relationship between the injury(s) and the symptom(s) exists.
(2) Patients with chronic oculomotor or vestibular disorders often recover in a few weeks with exercise-based therapeutic intervention (9–11). The brain injury model which has driven head trauma research for more than a century cannot account for this.

Several pathophysiological theories of concussion have been proposed and, for the most part, set aside. All but the reticular theory are entirely dependent on the presumption of a traumatic brain injury and all of them are primarily focused on loss of consciousness. A review of previous theories of concussion may be found in Supplementary material C.

Our difficulty in understanding concussion is surely at least partially due to the fact that our hypotheses are founded in the assumption of brain injury, an assumption that is verified in only 30% of those with mild TBI. Here we propose putative neurophysiological mechanisms which are founded in an assumption that is always true, viz. that atypical, high intensity, and often painful sensory stimuli directly excited the brain. Furthermore, in many patients, at least some of those stimuli persisted for days or weeks depending on soft tissue, skeletal, and other trauma coincident with the concussion. The proposed theory describes two sensory driven neurophysiological mechanisms which account for both short duration loss of consciousness and commonly seen acute, sub-acute, and chronic symptoms, oculomotor, vestibular, cognitive, emotional, and sleep dysfunction (12–15).

## Formulation and assertions of the theory

### Normal brain function relies on consistent patterns of tonic excitability across networked neuronal ensembles

We propose that the following model of sensory driven neurophysiological function underlies numerous complex brain functions including those which may become symptomatic after concussion, e.g., oculomotor, vestibular, attention, cognition, etc. These functions are due to the coordinated activity of one or more networks. Each network is composed of anatomically separate neuronal ensembles. Effective information transmission by a network of ensembles requires stable excitability for each. This ensures that the time of arrival of action potential volleys from each occurs when the receiving ensemble’s excitability cycle is at a receptive phase.

Neuronal ensembles are conceived as the functional units of the brain. Each subserves two functions, (i) information processing, i.e., recognition of the information contained in incoming (afferent) action potential volleys and transformation of that information to outgoing (efferent) action potential volleys and (ii) homeostatic, i.e., maintenance of a stable point about which the excitability of the ensemble cycles. This homeostatic function is necessary to effective reception, transformation, and transmission of information by the ensemble.

The population activity within a “source” ensemble transforms information in afferent action potential volleys. The transformed information is transmitted in efferent action potential volleys to “target” ensembles which, in turn, typically become sufficiently excited to themselves produce transformed information-carrying efferent volleys.

It is presumed that the information carried in the action potential volleys produced by a target ensemble is a complex transform due to both (i) the information encoded in the action potential volleys arriving from one or more source ensembles and (ii) the state of the information carrying activity within the target ensemble during reception of the afferent volleys.

If the target ensemble is already excited in response to previously received action potential volleys, the resultant efferent volleys may be modified in an immediate and associative fashion by the new afferent volleys. The particulars of this are beyond both the scope and the needs of the theory. The theory only asserts that the successful reception and transformation of incoming information to outgoing action potential volleys and the timing of those outgoing volleys depends on the level of excitability of the target ensemble and the phase in the ensemble’s cycle when the information-carrying action potential volleys arrive. The idea of a cycle comes from the presumption that many ensembles have a refractory period following the production of an outgoing action potential volley.

In order for a network as a whole to function normally, the information contained in the afferent action potential volleys to the network must be transformed and encoded in the efferent action potential volleys from the network. In particular, if the pattern of network tonic excitability results in discoordination, considerable afferent information may be lost, i.e., may not be represented in the efferent volleys.

For functions like memory and language production subserved by multiple large networks with each network comprised of many neuronal ensembles relatively widely separated in space and therefore time, we presume that there is considerable redundancy and that only a subset of the ensembles need be coordinated at any one moment. For functions like binocular eye movement and swallowing comprised of fewer neuronal ensembles narrowly separated in space and therefore time, we presume there is little or no redundancy and that the entire network must be consistently highly coordinated to function normally.

It is the dependence of both timing and successful information transmission on the dynamic level of ensemble excitability which is at the center of the theory of normal brain function. With a wave of incoming excitation, a neural ensemble reacts, i.e., it becomes sufficiently excited to produce transient information-carrying outflow which is then followed by a period of reduced excitability. The primary assertion of the theory is a homeostatic one, i.e., that the dynamic waxing and waning of a neural ensemble cycles around a stable point of tonic excitability. This homeostatic function is separate from but necessary to the information processing function of the ensemble.

### Assertions 1–4: normal/asymptomatic neurophysiological function

1) *Assertion*: The central excitability value about which a neuronal ensemble cycles, i.e., the “tonus” of the ensemble, is inherently stable. Under normal circumstances, changes in ensemble tonus occur slowly, i.e., over many months or years. See Supplementary materials B, B1 for experimental support for this assertion.
2) *Assertion*: Reliable function of a network relies on consistent timing of information transmission by its component ensembles. Timing of information transmission by a neuronal ensemble is dependent on the ensemble’s tonus, i.e., consistent timing depends on the stability of the ensemble’s tonic excitability. (16)
3) *Assertion*: The pattern of tonic excitability across the neuronal ensembles within a network is driven to change incrementally, typically over weeks or longer. Change occurs as the network repeatedly receives novel excitation or inhibition during transient periods when synaptic plasticity has been induced. For example, athletic practice may cause changes over relatively short periods. The alterations in skeletal motor coordination in a child in response to a growth spurt occur over much longer periods.
4) *Assertion*: The pattern of a network’s tonic excitability may change rapidly when the network repeatedly receives atypical excitation during a relatively brief period when synaptic plasticity is repeatedly induced.

### Neurophysiological mechanisms of concussion: brainstem crosstalk and alterations in regional tonic excitability

At the moment of impact to the head, high intensity sensory stimuli converge on the brainstem including one or more of the following:

- Loud sounds via the acoustic branch of cranial nerve VIII.
- Large and/or unusual head movements via the vestibular branch of cranial nerve VIII, and cervical C2-4.
- Intense pressure and pain to the face and/or scalp via cranial nerve V and cervical C2.
- High speed visual threats via the superior colliculus. This pathway is part of the pathway for eyeblink and other oculomotor reflexes mediated by the midbrain and brainstem.

Each sensory stimulus which arrives at the brainstem or midbrain stereotypically excites neural pathways which project widely throughout the brain including the cerebellum and the cortex. We conjecture that the convergence of high intensity sensory stimuli on the brainstem and midbrain can overwhelm the inhibitory mechanisms which segregate the activities of normally independent neural populations and, in so doing, produce incoherent crosstalk with atypical excitation or inhibition of cell populations which are normally uncoupled. One conjectured consequence is abnormal excitation of the vagal nucleus causing abnormal outflow on the vagus nerve. This putative pathological mechanism may cause outflow on the left vagus nerve resulting in profound bradycardia and contribute to loss of consciousness (17).

### The salience network and the locus coeruleus, putative origins for persistent neurologic and psychiatric post-traumatic symptoms

The salience network (SN), the default mode network (DMN), and the frontoparietal network (FPN) are three “canonical” functional neural networks whose interactions play a role in almost all cognitive functions. Each of the three has “… emerged from temporally organized coupling activity across vastly dispersed brain regions” based on fMRI studies (18). “The SN functions as a dynamic switch between concentration on self and the inner world, mediated by the DMN, and task-related and directed attention on outside stimuli maintained by the FPN.”

The SN is a widespread collection of brain regions including the cingulate and insular cortices, portions of the thalamus, several pontine nuclei, amygdala, and others. The insular cortex is an afferent hub which receives both interoceptive and visceroautonomic sensory information (19). The cingulate cortex is an efferent hub “responsible for generating relevant visceral, autonomic, behavioral, and cognitive responses” (20). The activity of the SN is involved in emotion, autonomic function, and self-awareness.

Volume loss or altered connectivity in SN regions has been linked to major depression, ADHD, anxiety, and others (20). The SN plays a central role in responding to both positive and negative “homeostatically relevant stimuli” including thirst, hunger, pain, bladder distention, embarrassment, amusement, compassion, tenderness, and humor (20).

The locus coeruleus (LC) is a bilateral pontine nucleus which is excited by trauma and other stressors (21, 22). Its connections to the SN are reciprocal and it is the major source of norepinephrine in the brain (23). LC neurons carry norepinephrine, and project widely throughout the brain. The LC does not behave as a uniform coordinated cell population. Its functional connectivity to the rest of the brain is systematically organized along its rostral/caudal axis (24). Its efferent anatomy is “modular … with segregated output channels and the potential for differential release and actions of norepinephrine upon its projection fields (25).” Those actions include selective cellular and behavior effects on stress responses, synaptic plasticity, learning, and memory (22).

In the moments following trauma with or without a blow to the head, the SN repeatedly excites the LC which induces synaptic plasticity widely in the brain. The SN also elevates arousal and engages the patient’s cognitive and emotional resources in attending to the trauma. What is “learned” by the brain in those moments? We assert that in a significant fraction of cases, it is altered tonic excitability patterns which approximate the current functional state of the brain. That state is one of high arousal due to excitation by high intensity atypical sensory stimuli reflective of the trauma, likely including both pain and fear. Those new excitability patterns may result in hypo or hyperexcitability and discoordination in some networks (assertion 2) with consequent symptoms. Variations in the alterations in tonic excitability patterns from one individual to another readily accounts for the differences in symptoms between concussions.

### Hyperexcitability of the locus coeruleus and other brainstem nuclei due to brainstem crosstalk

The predominance and intensity of cranial nerve sensory input during trauma with coincident concussion markedly elevates the chance that BCT will result and cause hyperexcitability of the LC. Such hyperexcitability could produce atypical LC output with a wider and less organized distribution since it would not respect the LC’s functional organization. Furthermore, since the LC is part of the ascending reticular activating system (ARAS) (26), other brainstem nuclei in the ARAS (25, 27, 28) may be atypically excited/inhibited. In addition, the left pontine nuclei adjacent to the LC where lesions are associated with coma (19) could be atypically excited or inhibited. Any of these may contribute to loss of consciousness and many of them may also produce atypical excitation of the salience network.

### Assertions 5–11: symptomatic neurophysiological function

5) *Assertion*: Immediate and transient brainstem crosstalk may be caused by the convergence of high intensity sensory stimulus at the moment of head impact.
6) *Assertion*: When it occurs, loss of consciousness (LOC) is due to (i) crosstalk-induced outflow on the left vagus nerve resulting in bradycardia and/or asystole and/or (ii) crosstalk induced atypical excitation or inhibition of the left pontine coma-specific brainstem nucleus (19) and/or (iii) atypical excitation or inhibition of the ascending reticular activating system, consistent with the reticular theory of concussion (25).
  Outflow on the left vagus nerve can cause bradycardia or asystole with consequent LOC (17). The plausibility of brainstem crosstalk as a contributor to LOC with concussion is supported by multiple reports that bradycardia and/or transient asystole have occurred with intraoperative stimulation or manipulation of the trigeminal nerve (29, 30), glossopharyngeal nerve (30), with tibial nerve stimulation (31) and with median nerve stimulation.^1^ The related trigeminal cardiac response/trigeminal depressor response (30, 32) is a well-documented surgical risk. In each of the cases referenced in these reports, the evoked bradycardia/asystole was blocked with the systemic administration of atropine, i.e., with blockage of acetylcholine, the neurotransmitter which mediates vagal suppression of the heart. As a side note, brainstem crosstalk may prove relevant to the neurophysiological mechanisms which underlie the neurovascular compression syndromes (33), e.g., trigeminal neuralgia and hemifacial spasm.
7) *Assertion*: When post-concussion brainstem mediated symptoms occur, viz. oculomotor or vestibular, brainstem crosstalk likely occurred with the concussion.
8) *Assertion*: Concussion with attendant trauma (i) repeatedly excites the salience network (SN) and therefore (ii) repeatedly excites the locus coeruleus (LC). The neurons of the LC (22) project widely throughout the brainstem, cerebellum, basal ganglia, and cerebral cortex (21, 34). Neuronal release of norepinephrine from LC neurons induces synaptic plasticity (21, 34, 35). These concurrent consequences of concussion may result in maladaptive alterations in tonic network excitability patterns, both within the SN, and much more widely due to the atypical sensory and SN excitation of much of the brain. Alterations in the tonic pattern of excitability within the SN itself may cause enhanced susceptibility with repeat concussion. MEG-derived evidence (36) for the involvement of the SN as a prime target for heightened excitability and as an intermediary in the genesis of secondary symptoms is detailed in Supplementary materials B, B2. In that work, SN regional excitability measures were prominent contributors to classifiers which distinguished between neurologically normal and chronically symptomatic patients with more than 95% accuracy. SN regions whose tonic neuroelectric activity measures contributed to the classifiers included the brainstem and bilateral anterior cingulate cortex, insular cortex, anterior thalamus, and nucleus accumbens.
  The SN responds to stimuli which are relevant to the individual’s homeostasis/survival (20) including social context (37) and sense of well-being (38). This mechanism may therefore be applicable to other clinical entities, e.g., depression or PTSD in first responders (39, 40) and those exposed to traumatic events (41), and the high incidence of post-concussion-like symptoms in those without history of concussion (42).
  We conjecture that the pattern of neuronal ensemble excitability within a network is regulated by subsets of the ensemble populations. It is persistent alterations in that regulation produced by concussion which changes the pattern of tonus and causes network discoordination with resultant symptoms. Though the changes in the pattern of tonus may fall within the range of normal, the new pattern must be adjusted/altered to establish normal coordination. Just as physical coordination deteriorates in children during a growth spurt and then improves with time and practice, concussion may produce loss of coordination degrading information transmission and cognitive function which progressively resolves with learning.
  That learning requires (i) the exercise of sensing and responding to the world as did establishment of the original pattern and (ii) synaptic plasticity coincident with the exercise to enable alterations in tonus. These two requirements for recovery correspond particularly well to the established treatments for oculomotor (11, 43, 44) and vestibular (45, 46) symptoms. These treatments are stressful since they aggravate the targeted symptoms. But that stress promotes the efficacy of the exercise by exciting the locus coeruleus which induces synaptic plasticity. The conjectured necessity of synaptic plasticity coincident with exercise for effective treatment readily accounts for the fact that vestibular and oculomotor treatments which evoke symptoms produce recovery in most patients whereas symptom avoidance therapies do not. (46).
8) *Assertion*: With brainstem crosstalk-induced locus coeruleus hyperexcitability, the resultant widespread and atypical pattern of synaptic plasticity will enhance the development of maladaptive patterns of network excitability throughout the brain. This may account for the high incidence of depression and PTSD in concussion compared with trauma without concussion (47–49).
9) *Assertion*: Symptomatic patterns of tonic excitability in small-scale short latency networks return to their pre-concussion patterns with symptom recovery, e.g., those which underlie oculomotor and vestibular function. Symptomatic patterns of tonic excitability in large-scale, long-latency networks altered by concussion do not, in general, return to their pre-concussion patterns with symptom recovery, e.g., networks which underlie cognition and mood. This assertion is supported by the magnetoencephalographic findings detailed in Supplementary materials B1, B2. In brief, the classifiers trained on the baseline neurologically normal vs. chronically symptomatic concussion patients classify the follow-up recordings with more than 90% accuracy, even though many of the concussion patients recovered in the 6-month interim.
10) *Assertion*: For chronic symptoms, three mechanisms are proposed. Note: secondary symptoms are those which spontaneously resolve with treatment of primary symptoms.

a) *Primary symptoms* are caused by network discoordination due to anatomic or cytoskeletal axonal injury. This is currently the presumed cause of all post-concussion symptoms.
b) *Primary symptoms* are caused by network discoordination due to altered tonic excitability regardless of anatomic or cytoskeletal neuronal injury. Symptom-dependent network discoordination may manifest itself as hypo or hyperexcitability of one or more neuronal ensembles.
c) *Secondary symptoms* are caused by transient discoordination in networks which repeatedly receive atypical patterns of excitation from primary symptomatic networks but do not receive sufficient locus coeruleus input to become plastic. Hence the tonic pattern of excitability for a “secondary symptom” network is not altered, so when the abnormality in the “primary symptom” network is resolved, the secondary symptom(s) also resolve.

## Consequences and tests of the theory

### Consequences of the theory (Figure 1)

**Figure 1:**
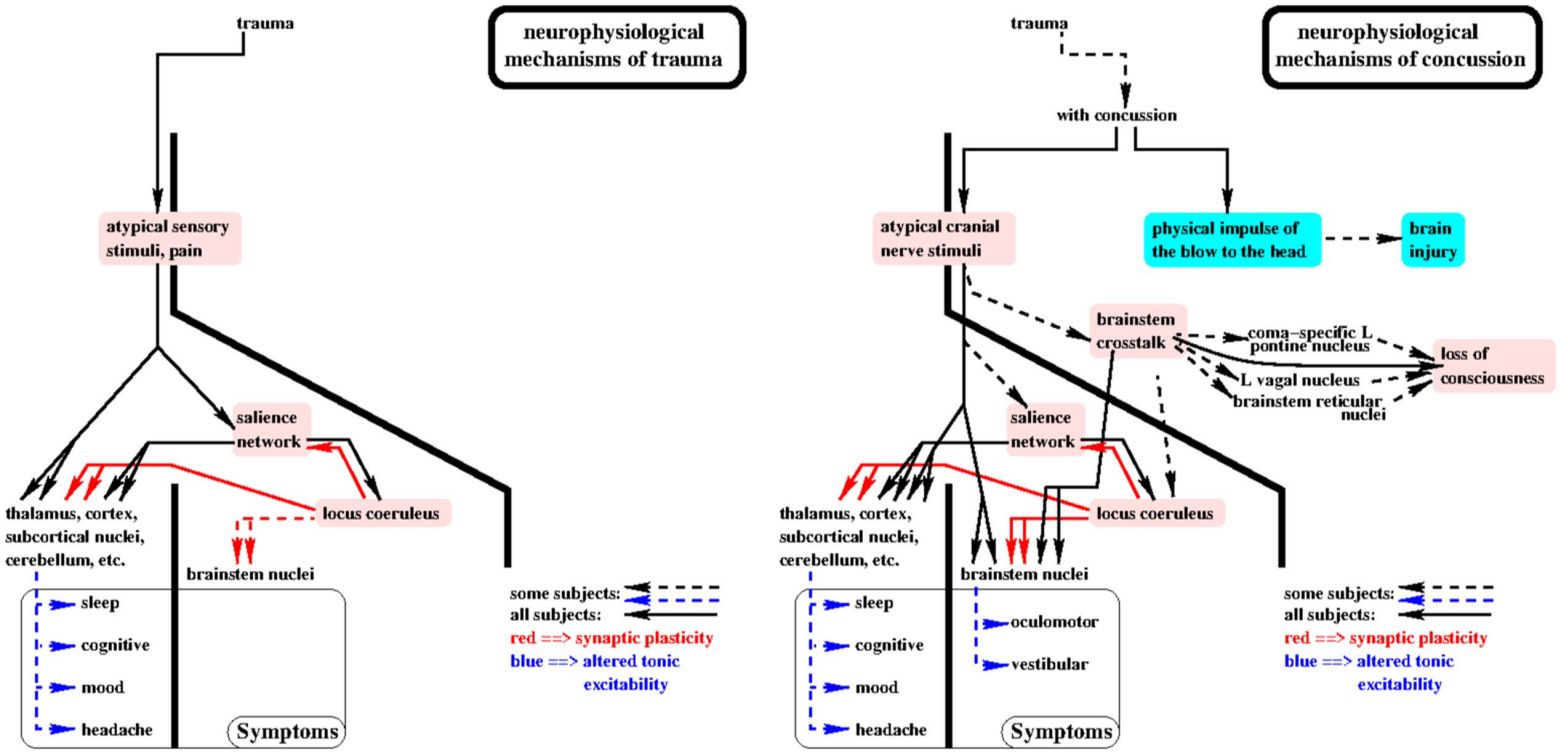
Consequences of the theory are shown in this diagram of the putative sensory driven neurophysiological mechanisms by which trauma (left panel) and concussion (right panel) cause symptoms. The primary elements of the theory are highlighted in pink. Solid lines with arrowheads indicate cause/effect relationships which putatively occur in all subjects. For example, when loss of consciousness or post-concussion oculomotor or vestibular symptoms occur, then brainstem crosstalk occurred at the moment of concussion. See “Consequences of the theory” for more details.

(1) If loss of consciousness (LOC) occurred with the concussion, then brainstem crosstalk (assertion 6) occurred. For short duration LOC, i.e., less than 90 s, the vagal mechanism may be solely responsible.
(2) For longer duration LOC, the coma-specific mechanism (b) must be involved since vagal bradycardia sufficient to cause LOC for more than 90 s would likely result in an irreversible ischemic insult. In consideration of this point, it is possible that the brainstem crosstalk mechanism for LOC proposed here may also contribute to sudden infant death syndrome (SIDS) in some infants. This comports with the intuitive notion that the immature brainstem is susceptible to brainstem crosstalk: paroxysmal vagal overactivity has been considered as a plausible cause of SIDS and infants at risk for SIDS have shown brief episodes of bradycardia in response to digestive, nasopharyngeal, and laryngeal stimulation, gastroesophageal reflux, and fear (50).
(3) If significant trauma occurred at the time of concussion, then locus coeruleus (LC) and salience network (SN) excitation (assertion 7) also occurred at that moment and likely repeatedly for days due to persistent traumatic discomfort and pain.
(4) If both loss of consciousness and significant trauma occurred with the concussion, then the effects of repeated LC and SN excitation would more likely result in widespread alterations in tonic excitability with greater likelihood and severity of multiple symptoms.
(5) SN-LC excitation occurs with any trauma. Brainstem crosstalk (BCT) and high intensity cranial nerve sensory input only occur with concussion, hence brainstem-mediated symptoms only occur with concussion. If post-concussion oculomotor or vestibular symptoms occur, then BCT likely occurred with the concussion.
(6) If loss of consciousness occurred, i.e., brainstem crosstalk (BCT) occurred at the time of concussion, BCT may persist and is more likely to occur in the future.
(7) If BCT occurred,

(a) symptomatic alterations in tonic excitability of brainstem mediated functions are more likely, e.g., oculomotor and vestibular, and
(b) asymptomatic/subclinical alterations in tonic excitability of brainstem mediated functions are likely, e.g., perturbation in the coordination of eyeblink, swallowing, phonation, etc.

(8) In concussion patients with multiple symptoms, targeted treatment for one presumed “primary” symptom at a time with continuous tracking of all symptoms will reveal which underlying networks display learned dysfunctional excitability patterns, causing their primary symptoms. These are the symptoms which require treatment. Secondary symptoms, i.e., those which resolve with successful treatment of primary symptoms, are those which arise from networks which are driven into dysfunctional excitability patterns by primary symptom networks, but which never “learned” those dysfunctional patterns.
(9) Avoidance of symptom provocation during treatment degrades the effectiveness of the treatment because it reduces the evocation of synaptic plasticity by locus coeruleus excitation and therefore disables learning.

### Tests of the theory

The theory proposes two mechanisms by which sensory input at the moment of head trauma causes symptoms. One of these mechanisms involves the salience network and the locus coeruleus and is presumed to be activated by any trauma. The other, which is due to brainstem crosstalk (BCT), is specific to head trauma. The primary focus of the proposed tests of the theory is on the acute and sub-acute consequences of BCT. The reasons for this focus are as follows. (i) The role of BCT is central to the proposed theory and is particular to head trauma. If tests for BCT fail, the theory fails. But if some of the tests for BCT demonstrate that it had occurred, that will provide substantive support for the validity of the theory. (ii) The BCT assertion is readily falsifiable by testing for a variety of subclinical functional perturbations in the acute and sub-acute periods following injury. All of the proposed tests require repeated measures from each subject over a short period ranging from a few hours to 6 weeks. This will minimize loss to follow-up and expense. (iii) Numerous research groups already have the requisite expertise, equipment, and subject populations to run one or more of the proposed tests with modest effort and expense.

For those in whom BCT occurred, there is no reason to think that this cause for symptoms was confined only to the neuronal ensembles in the networks which subserve functions which were symptomatic. On the contrary, we expect to find other signs of BCT, e.g., elevated oculocardiac reflex (51, 52), and subclinical indications of altered tonic excitability in other brainstem mediated systems, e.g., altered split-second timing in the blink reflex, swallowing, phonation, and facial movement. See Supplementary material A for background and measurement capabilities for these functions.

A set of tests is therefore proposed for the presence of subclinical perturbations in these functions. The incidence of such subclinical findings is expected to be greatest in those with history of concussion and with indicators of BCT, viz. loss of consciousness and/or oculomotor or vestibular symptoms. But subclinical findings may also be found with less frequency in those without history indicative of BCT. If found, this would show that BCT had occurred, but had not affected consciousness, oculomotor, or vestibular function sufficiently to be detected.

In those with symptoms indicative of BCT, subclinical findings should change with symptom resolution. This prediction would be most readily tested in short term longitudinal studies of those in treatment for oculomotor or vestibular disorders, since these are successfully treated in a few weeks in most cases (9, 10, 45). Given the large between-subjects differences in the concussion patient population, a longitudinal study will maximize the statistical power of the tests. In particular, the repeated measures from each individual will maximize the confidence that can be placed in the findings, should they fail to falsify the theory.

Given these consideration, tests are proposed in two tiers. The first tier tests are those with the greatest sensitivity to BCT. Hence, these are the tests which, if they fail to demonstrate BCT, will provide the greatest confidence in the falsehood of the theory. Their key characteristics are as follows.

(1) A cohort is selected with clinical history indicative of BCT, viz. loss of consciousness and/or current symptoms of oculomotor and/or vestibular dysfunction.
(2) Repeated measures of one or more of the following are obtained: oculocardiac reflex, split second timing of conjugate eye movements, blinking, phonation, swallowing, and/or facial movements. See Supplementary material A for a brief review of high precision measures of these functions. The first measures should be obtained as soon after presentation as possible while the patient is symptomatic. Repeated measures should be continued for at least 1 month or, for those in treatment, for at least 1 month following the beginning of treatment. Since no baseline pre-concussion measures will have been obtained, measures must be found which change as symptoms resolve to infer that the changing measures reflect the presence and subsequent decrease in BCT. The use of timing measures is based on the theoretic assertion that alterations in the pattern of network excitability cause alterations in timing. Hence, findings of coincident resolution of symptoms with coincident changes in timing supports both assertions, viz. that functional timing depends on network excitability and that BCT was present.

The first tier tests may also prove conclusive when administered to those with history of concussion who did not lose consciousness or have post-concussion oculomotor or vestibular symptoms. Though their history does not include the signature indicators of BCT, the fact that they were sufficiently symptomatic to carry the diagnosis of concussion is sufficient to hypothesize that some may display subclinical manifestations of BCT. These are second tier tests. The likelihood of detecting BCT in these subjects is less than in those with symptoms suggesting BCT, but the BCT tests in this population are additional bona fide tests of the theory.

Athletes engaged in high impact sports may sustain subclinical blows to the head which produce BCT with consequent measurable perturbations in the same functions tested in tier one. Comparable sensory input and therefore BCT with comparable effects may also be found in some dental patients undergoing anxiety-inducing or painful procedures which include drilling or scraping, e.g., root canal, deep cleaning. Tests for oculocardiac reflex or split second timing changes in brainstem-mediated functions in these subject populations comprise additional tier two tests of the theory. For these tests, comparisons would be made between baseline and short term follow-up measures, e.g., pregame and postgame or pre- and post-dental procedure.

In the athletic cohorts, continuous heart rate could be monitored along with head acceleration. Consistent reduction in heart rate immediately following significant head acceleration would indicate that BCT had occurred and had produced atypical outflow on the left vagus nerve. For interior offensive linemen in American football, 20 or more contacts would be expected per game for each player. This would provide considerable statistical power to detect or conversely to rule out BCT. Hence it would constitute a relatively strong test of the theory.

Finally, transient reduction in heart rate following palpable electrical stimulation of the face (trigeminal nerve) or scalp (cervical C2) would provide direct evidence for BCT. This simple measure would be most likely to succeed in concussed individuals with indications that BCT had occurred, viz. loss of consciousness, oculomotor or vestibular dysfunction.

## Discussion

For more than a century, our understanding of concussion has been based on two restrictive assumptions: (i) The pathophysiological agent of concussion is the physical impulse delivered by the blow to the head. (ii) Post-concussion symptoms are due to brain injury. The tight linkage of the two assumptions is forced, i.e., that brain injury was caused by the blow.

In the theory proposed here, assumption (i) is relaxed to allow for the pathophysiological agency of atypical, high intensity, and often painful sensory input to the nervous system at the moment of concussion and, in most cases, continuing for minutes, days, or weeks afterward. This employs the hitherto omitted facts that there is a sensory component to every head trauma and that sensory input directly stimulates the brain.

The mechanisms proposed in the theory account for the symptoms of concussion and much of what is known about symptom recovery. They do so without relying on brain injury as the cause. That accounts for the fact that no objective evidence of brain injury is found in 75% of those with concussion. It is true that objective evidence of brain injury is found in about 25% of those with concussion and in all of those with moderate or severe head trauma, i.e., about 40% of all comers with head trauma. Yet even in those patients, the proposed sensory induced mechanisms may also contribute to their symptoms.

With 40 years of powerful and constantly evolving brain imaging and biochemical methods, no brain injury is identifiable in fully 60% of all patients with head trauma. That preponderance of negative findings provides considerable evidence that both long-standing assumptions on which our limited understanding of concussion is based are false for the great majority of concussion patients.

Here are the key elements of the theory: Two potentially symptom-initiating events coincide with concussion: (i) a blow to the head and (ii) atypical, high intensity sensory stimuli, often including pain. These initiating events can cause persistent dysfunctional alterations in the stable patterns of network excitability on which symptom-free neurological function depends via three mechanisms:

(1) Brain injury caused by the physical impulse of the blow can affect network excitability by directly or indirectly damaging neuronal cell bodies and/or axons.
(2) Sensory stimuli coincident with concussion induce widespread plasticity in the brain by exciting the salience network which, in turn, excites the locus coeruleus. Continued discomfort due to residual injury from trauma can repeatedly excite the salience network and locus coeruleus for minutes, days, or weeks, repeatedly inducing plasticity and enabling neurophysiological “learning” widely in the brain. This mechanism enables “learning” of dysfunctional patterns of network excitability, and causes acute, subacute, and chronic symptoms.
(3) Convergence of atypical, high intensity stimuli on the trigeminal and other brainstem nuclei can cause brainstem crosstalk (BCT). BCT is hypothesized to occur when neuronal ensembles in the brainstem or midbrain which are normally independent excite each other. This can cause loss of consciousness and can alter the coordination within and between neuronal ensembles which mediates oculomotor and vestibular function sufficiently to cause symptoms.

The theory is parsimonious in that it relies on just a few elements. These are the high intensity and often painful sensory input both at the moment of the blow to the head and repeating in the minutes, days, or weeks afterward; the roles of the salience network and the locus coeruleus in inducing plasticity and neurophysiological “learning” in the brain; and the roles of the brainstem nuclei affected by BCT.

The theory posits two putative mechanisms. (i) The pattern of excitability about which a network of neuronal ensembles fluctuates is inherently stable, typically over years. Rapid changes in that pattern produce symptoms in the function which the network underlies. (ii) Brainstem crosstalk produced by the convergence of atypical, high intensity sensory stimuli on the brainstem is the cause of both loss of consciousness and oculomotor and vestibular dysfunction, i.e., the symptoms which are specific to head trauma. Mechanism (i) relies only on trauma which produces painful, high intensity sensory input which repeatedly excites the salience network and locus coeruleus. This mechanism is not specific to concussion and may, if true, prove useful in understanding the neurological sequelae of trauma in general.

The theory provides causal neurophysiological mechanisms for the following:

(1) A blow to the head can cause transient loss of consciousness. Per the theory, this is due to brainstem crosstalk stimulation of the left vagal nucleus and/or the left pontine coma-specific nucleus, and or the brainstem nuclei in the ascending reticular activating system.
(2) Symptoms in brainstem mediated functions, i.e., oculomotor and vestibular, occur in head trauma but not in trauma in general. Per the theory, these symptoms are due to high intensity cranial nerve sensory stimulation and likely brainstem crosstalk, both of which are specific to concussion.
(3) Rapid recovery, viz. a few weeks, occurs in most patients with oculomotor or vestibular symptoms. Per the theory, there is no injury to the involved brainstem networks in those who recover acutely or in weeks with therapy. Instead, these closely separated short latency networks return to their original patterns of tonic excitability.
(4) Per the theory, acute and chronic symptoms in functions mediated by distributed cortical/subcortical networks, e.g., mood, cognition, are due to altered patterns of excitability in these long latency networks. Recovery from such symptoms sometimes requires treatment for them. Sometimes they resolve coincident with treatment of other symptoms, particularly those which are brainstem mediated.

a If the altered patterns were learned, i.e., the alterations occurred in the acute phase after concussion due to locus coeruleus-induced synaptic plasticity, then the symptoms are primary and must be treated to resolve.
b If the altered patterns were not learned, but instead are *driven* by input from networks whose activity underlies primary symptoms, then the symptoms are secondary and will resolve when the primary symptom(s) resolve.
c An altered tonic pattern of excitability within the salience network may lead to enhanced symptoms with a repeat concussion.

(5) Exercise therapies which evoke symptoms enable recovery whereas symptom avoidance strategies do not.
(6) Network excitability patterns derived from magnetoencephalography demonstrate (i) high long-term test–retest reliability (see Supplementary material B1), (ii) distinguish with high accuracy between those without history of concussion and those with concussion and chronic symptoms (see Supplementary material B2), and (iii) distinguish those with and without individual symptoms in chronic concussion patients with multiple symptoms (see Supplementary material B3).
(7) The symptoms caused by head trauma do not depend on brain injury.

We propose numerous tests of the theory. No one test by itself can fully falsify the theory. That is because we can infer no *a priori* expected incidence of the phenomena proposed for measurement. For some of those phenomena, the true incidence may indeed be near zero. However, if many of the tests fail to produce findings consistent with the assertions of the theory, then the theory must be false. On the other hand, if some of the tests yield positive findings for even a modest percentage of subjects, that will provide substantive support for the validity of the theory.

Many of the phenomena are readily measurable with relatively inexpensive equipment to which many laboratories already have access and considerable expertise. We hope that those with the wherewithal will choose to run tests of the theory.

## Data Availability

Publicly available datasets were analyzed in this study. This data can be found at: https://cam-can.mrc-cbu.cam.ac.uk/dataset/, Cambridge Centre for Ageing and Neuroscience Data Access Portal.

## Footnotes

^1^Asystole was observed by one of us (DK) for at least 15 s on five occasions during retro mastoid craniectomies due to direct manipulation of the trigeminal nerve, on two occasion during cervical spine surgeries due to median nerve stimulation, and once during lumbar spine surgery due to tibial nerve stimulation. In each case, administration of atropine prevented further episodes.

## Appendix: glossary and abbreviations

**Acetylcholine (ACh)** is a neurotransmitter found in brainstem nuclei which depress/inhibit other neuronal structures. Release of ACh from injured brainstem neurons is central to the pontine cholinergic system hypothesis.

**Action potential**

Sensory induced **brainstem crosstalk (BCT)** is a putative neurophysiological mechanism whereby neuronal ensembles in the brainstem or midbrain which are normally independent excite each other. The theory asserts that this occurs in some patients due to the atypical multisensory input convergent on the brainstem at the moment of concussion.

**Ascending reticular activating system** (**ARAS**) (26)

**Computed tomography (CT)**

**Diadochokinetic** tasks test the ability to repeat rapidly alternating syllables, e.g., “puh tuh kuh” (53, 54).

The **Glasgow Coma Score (GCS)** is a widely used clinical rating scale which quantifies the severity of a concussion. At least 30 min must have elapsed following the blow to the head to insure validity of the assessment. It ranges from 3 to 8 (severe TBI), 9–12 (moderate TBI), and 13–15 (mild TBI) (1).

**Head trauma** is a blow to the head. Head trauma is a catch-all term which applies to concussion, mild TBI, or TBI in general.

- **Traumatic brain injury (TBI)** is a documented blow to the head for which evidence of brain dysfunction or injury was witnessed or found on clinical exam, neuroimaging, or biochemical assay.
- **mild TBI** (**mTBI**) is a TBI for which GCS on clinical exam is 13–15.
- As used in this paper, **concussion** is a blow to the head which fulfills the criteria for mild TBI, i.e., GCS = 13–15. However, the term “concussion” does not imply that the patient suffered a brain injury.

The **locus coeruleus** (**LC**) is a bilateral pontine nucleus which is excited by trauma and other stressors (21, 22).

**Loss of consciousness (LOC)**

**Magnetic resonance imaging (MRI), functional MRI (fMRI)**

**Magnetoencephalography** (**MEG**)

A **neuronal ensemble** is conceived as the primary functional unit in the hierarchical organization of the brain. It a collection of neurons which are more tightly neuroelectrically coupled to each other than to neurons outside the ensemble. The activity of the neurons within an ensemble is cooperative in subserving the functions of recognizing the input to the ensemble, transforming it, and neuroelectrically projecting the transformed output to other ensembles.

A **neuronal network** is conceived as the secondary functional unit in the hierarchical organization of the brain. It is a collection of neuronal ensembles which are more tightly neuroelectrically coupled to each other than to ensembles outside the network. The salience network is an example.

**Nucleus –** functionally/anatomically distinct collection of neurons.

**Oculocardiac reflex (OCR)**

**Phonation** is the production and utterance of speech sounds.

**Post-traumatic stress disorder (PTSD)**

The **salience network** (**SN**) is a neuronal network spanning a widespread collection of brain regions including the cingulate and insular cortices, thalamus, several pontine nuclei, and the amygdala. The activity of the SN is involved in emotion, autonomic function, and self-awareness. The SN plays a central role in responding to both positive and negative “homeostatically relevant stimuli” including thirst, hunger, pain, bladder distention, embarrassment, amusement, compassion, tenderness, and humor (20). The SN, the default mode network (DMN), and the frontoparietal network (FPN) are three “canonical” functional neural networks whose interactions play a role in almost all cognitive functions.

**Sudden Infant Death Syndrome (SIDS)**
