# Supplementary material for: Sensory driven neurophysiological mechanisms of concussion: a parsimonious and falsifiable theory
Source: Front Neurol. 2025 Apr 30;16:1547786. doi: 10.3389/fneur.2025.1547786 (PMC12074929; doi:10.3389/fneur.2025.1547786)
Supplement: Supplementary file 1 [file Supplementary_file_1.docx]

[Supplement A: High precision measures of asymptomatic brainstem mediated functions 1](#_Toc195802744)

[Oculocardiac reflex (OCR) 1](#_Toc195802745)

[Coordinated eye movements 1](#_Toc195802746)

[Blinking 2](#_Toc195802747)

[Speech production: phonation 2](#_Toc195802748)

[Swallowing 2](#_Toc195802749)

[Facial movements 2](#_Toc195802750)

[Supplement B: MEG-derived patterns of tonic regional excitability 2](#_Toc195802751)

[B1. Test-retest reliability 3](#_Toc195802752)

[B2. Classification of those with and without history of concussion 5](#_Toc195802753)

[B3. Classification of the concussed with and without symptoms 7](#_Toc195802754)

[Supplement C: Theories of concussion 7](#_Toc195802755)

[Combined biochemical/physiological theories. 7](#_Toc195802756)

[Biochemical mechanisms 8](#_Toc195802757)

[Biomechanical mechanisms 9](#_Toc195802758)

[References 10](#_Toc195802759)

## Supplement A: High precision measures of asymptomatic brainstem mediated functions

Per the theory, the tonic excitability and therefore the timing of all of the brainstem nuclei engaged in the following functions are susceptible to alteration by concussion, concussion-induced BCT, and recovery from concussion.

### Oculocardiac reflex (OCR)

The OCR is trigeminal-vagal induced bradycardia typically resulting from compression of the eye or retraction of ocular muscles (1). It is seen in approximately 30% of eye surgeries and produces profound bradycardia in nearly 10% of strabismus cases (2). Elevated OCR with significantly increased bradycardia has been found in both infants with near miss for sudden infant death syndrome (SIDS) and in their siblings (3).

### Coordinated eye movements

Routine vestibular-ocular motor screening (VOMS) includes physical examination by a trained clinician of ocular smooth pursuit, horizontal and vertical saccades, horizontal and vertical vestibular ocular reflex, and visual motion sensitivity (4): Table 3. These measures are sensitive to recovery from post-concussion vestibulo-ocular symptoms (4-6). The extraocular muscles which enable both binocular eye movements (7) and the vestibulo-optic reflex (8) are coordinated and driven by brainstem nuclei. There are also numerous brainstem nuclei which function as oculomotor neural integrators (9). Eye movements may be sampled photographically at up to 1200 hz (10,11) with high angular precision.

### Blinking

A blink may be elicited by volition or reflexively by sensory input via photic stimulation (optic nerve), sound (cochlear nerve), head movement (vestibular nerve), a touch or air puff to the cornea, facial or mouth pain (trigeminal nerve), or via median or other somatosensory nerve (12), Fig 1. These sensory stimuli pass through the midbrain (visual) or the pons (all others) and excite output on the facial nerve (close eye) and then the oculomotor nerve (open eye). Blink reflex timing is sensitive to age (13), exertion (14,15), and concussion (14,16). Blink reflex parameters are readily measurable with 4 msec precision (17).

### Speech production: phonation

Numerous biomarkers of concussion have been found in cross-sectional studies of speech production in young athletes using DDK tasks (18,19). Much of the coordinating functionality of speech production and the final motor neurons which innervate the organs of speech production including the larynx, tongue, lips, masseter, and diaphragm all reside in brainstem nuclei (20-22). High precision acoustic metrics of phonation are readily obtainable using computer-assisted analysis of audio recordings (23,24).

### Swallowing

Like speech, much of the coordinating functionality and also the final motor neurons which innervate the organs of swallowing reside in the brainstem (25). Split second timing is noninvasively measurable using sound sensors, electromyography, ultrasound, and others (26).

### Facial movements

Much of the coordinating functionality and also the final motor neurons which innervate the facial muscles reside in the brainstem. Measurement of facial movement is noninvasively measurable with high precision using high speed computer-assisted videography (27,28).

## Supplement B: MEG-derived patterns of tonic regional excitability

With a wave of incoming excitation, a neural population reacts, i.e. it becomes sufficiently excited to produce transient coherent outflow which is then followed by a brief period of reduced excitation. The dynamic waxing and waning of a neural population varies around a relatively stable point, the “tonus” of the population. This assertion is the starting point for the theory. It is supported by work with magnetoencephalographic (MEG) recordings (29) which demonstrated high long-term test-retest reliability (see supplement C1) in both a neurologically normal cohort (CamCAN), and chronic concussed cohort (TEAM-TBI).

For both cohorts, high resolution MR imaging, diffusion-weighted MR imaging, and both resting and task magnetoencephalographic recordings were obtained at baseline and at follow-up, CamCAN (*mean* 16.1 months), TEAM-TBI (*mean* 6.1 months). The CamCAN cohort spans ages 18-87 and included 606 subjects at baseline and 213 at follow-up. The TEAM-TBI cohort spans ages 20-63 and includes 60 subjects at baseline and 37 at follow-up. In addition to comprehensive neuroimaging, these volunteers with chronic high symptom load underwent clinical testing, and were provided with conservative therapies for oculomotor, vestibular, sleep, and cognitive symptoms.

151 stereotypic cortical, adjacent white matter, and subcortical regions and 18 deep white matter tracts were identified from the MR imaging in each subject using Freesurfer 5.3 (30,31) and Tracula 1.22 (32) respectively. A single numeric measure of tonic neuroelectric activity was then extracted from each 5-10 minute human magnetoencephalographic recording (29). Neuroelectric currents were identified, validated (p < 10^-12^ for each), and counted within each region. The count for each subject x region was reduced to a density, i.e. a count per unit volume, and then normalized to dimensionless number with a *mean* of 1.0 by dividing the regional density by the total density for the subject’s brain as a whole.

### B1. Test-retest reliability

Regional measures for baseline resting vs task recordings were compared using Pearson correlation to measure test-retest reliability, both short-term, ~30 minutes, and long-term, *mean* 16.1 months for CamCAN, *mean* 6.1 months for TEAM-TBI. The correlations were computed for each subject across the 169 regions for that subject. Hence the correlations measure the stability of the pattern of tonic regional excitability across the brain of an individual. Control between-subjects correlation was computed for each subject’s 169 regional measures vs the regional measures of a randomly chosen subject.

|  | CamCAN | TEAM-TBI |
| --- | --- | --- |
| Short-Term Test-Retest Correlation | 0.906 (*sd*: 0.072; *n* = 606) | 0.954 (*sd*: 0.043; *n* = 62) |
| Long-Term Test-Retest Correlation | 0.804 (*sd*: 0.120; *n* = 213) | 0.886 (*sd*: 0.071; *n* = 37) |
| Between-Subjects Correlation (Control) | 0.341 (*sd*: 0.162; *n* = 606) | 0.450 (*sd*: 0.151; *n* = 62) |
| Table B1. For both cohorts, the brain-wide pattern of regional excitability demonstrated unprecedented test-retest reliability. The short-term correlation is greater than the long-term correlation for the CamCAN cohort (*Welch’s t* = 11.7; *df* = 267; *p* << 10^-6^ ) and for the TEAM-TBI cohort (*Welch’s t* = 5.27; *df* = 52; *p* = 10^-6^ ). The between-subjects correlation is greater for the TEAM-TBI cohort than for the CamCAN cohort (*Welch’s t* = 5.38; *df* = 76; *p* << 10^-6^ ) | | |

As shown in Table B1, both the short-term and long-term test-retest reliability of these neuroelectric measures are considerably higher than has been reported in other MEG or fMRI studies (29), section 4.2, due to the unprecedented precision with which the measures were obtained. The high long-term test-retest reliability found with these MEG-derived neuroelectric measures comports with theoretic assertion (1): “The central excitability value about which a neuronal ensemble fluctuates, i.e. the “tonus” of the ensemble, is inherently stable.” The even higher short-term reliability also comports with assertion (1), since it was measured by computing the correlation between resting and task recordings obtained in the same sitting. Hence there is little difference in regional tonus depending on brain state, i.e. rest vs task in this case.

| 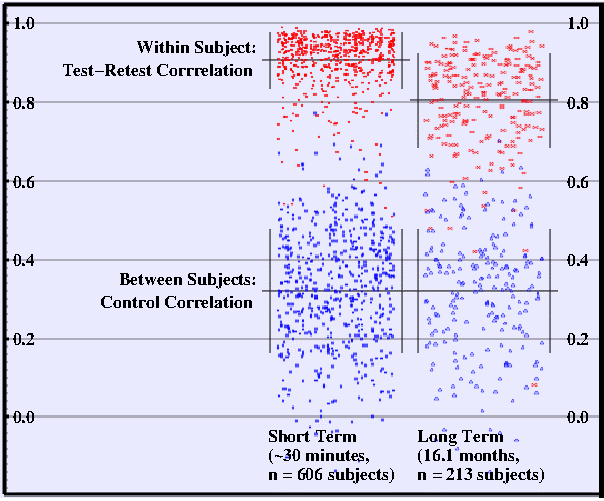 |
| --- |
| Figure B1. **Test-retest reliability for the CamCAN cohort.** Both short term (~30 minutes) and long term (*mean* 16.1 months) test-retest reliability are shown in red for the pattern of tonic excitability of each subject. Each dot represents the correlation over all 169 regions for a single subject. Short term test-retest correlations are high. This shows that tonic regional excitability is independent of brain state since these measures were compared between resting and task recordings obtained in the same sitting. Long term test-retest correlations are only slightly lower. The between-subject (control) correlations shown in blue are low, demonstrating that the patterns of tonic regional excitability across the brain are different from one subject to the other. For each set of correlations, the *mean* across all subjects is shown (horizontal line) along with ±1 *sd* vertical error bars. |

The *mean* between-subjects (control) correlation was much lower than the within-subjects correlation (test-retest reliability). Hence the differences in the regional tonus patterns between individuals are much greater than those seen between an individual and themself many months later. Another way to say this is that the commonality in the pattern of regional tonus across each cohort is much less than the persistence of the specific pattern seen within each individual.

The between-subjects correlation is significantly higher for the TEAM-TBI cohort than for the CamCAN cohort. Hence the commonality in the pattern of regional tonus across the TEAM-TBI cohort is significantly greater than that for the CamCAN cohort.

The classification was accurate for almost all the TEAM-TBI cohort members at follow-up, in spite of the fact that half had significantly recovered from at some of their symptoms. These quantitative findings comport with the assertion that the excitability patterns in the salience network and perhaps others were altered at the time of the concussion and did not return to their original state with recovery. Prominent contributors to the classifier included many salience network regions, e.g. anterior cingulate, insula, thalamus, brainstem, and the nucleus accumbens.

Each of the brain regions identified by Freesurfer and Tracula presumably covers many of the neuronal ensembles referred to in the theory, i.e. collections of neurons which function as nodes in a network which subserves a function which may become symptomatic. Depending on its volume, each such region presumably covers at least a few neuronal ensembles and for larger regions, many. Hence the consistently high long term test retest reliability found in this work is interpretable as an average property over many ensembles. The findings therefore support theoretic assertion (1) with the caveat that a measure of central tendency is inherently more stable than its components.

### B2. Classification of those with and without history of concussion

MEG-derived patterns of regional excitability were tested for their ability to classify each subject into their cohort using stepwise linear discriminant analysis (33). The data were divided into disjoint training and test sets to insure validity of the results. The classifier was trained on the baseline resting recordings and then tested on the baseline task and follow-up resting recordings of both cohorts (see Table B2).

Classification accuracy was greater than 90% for all of the groups including the follow-up TEAM-TBI group, in spite of the fact that half had significantly recovered from at least some of their symptoms. These quantitative findings comport with assertion (8), that the excitability patterns in the salience network and perhaps others were altered at the time of the concussion and per assertion (10), did not return to their original state with recovery. Prominent contributors to the classifier included many salience network regions, e.g. anterior cingulate, insula, thalamus, brainstem, and accumbens.

| **Classification Accuracy** | | correct / *n* | % correct | *p-value* |
| --- | --- | --- | --- | --- |
| Training Sets  (Jackknifed Classification) | CamCAN: baseline rest | 603 / 613 | 98.4% | 10^-163^ |
|  | TEAM-TBI: baseline rest | 59 / 62 | 95.2% | 10^-14^ |
| Test Sets | CamCAN: baseline task | 595 / 606 | 98.2% | 10^-159^ |
|  | CamCAN: follow-up rest | 209 / 213 | 98.1% | 10^-56^ |
|  | TEAM-TBI: baseline task | 60 / 62 | 96.8% | 10^-15^ |
|  | TEAM-TBI: follow-up rest | 35 / 38 | 92.1% | 10^-7^ |
| Table B2. Classification of those with and without history of concussion. | | | | |

| 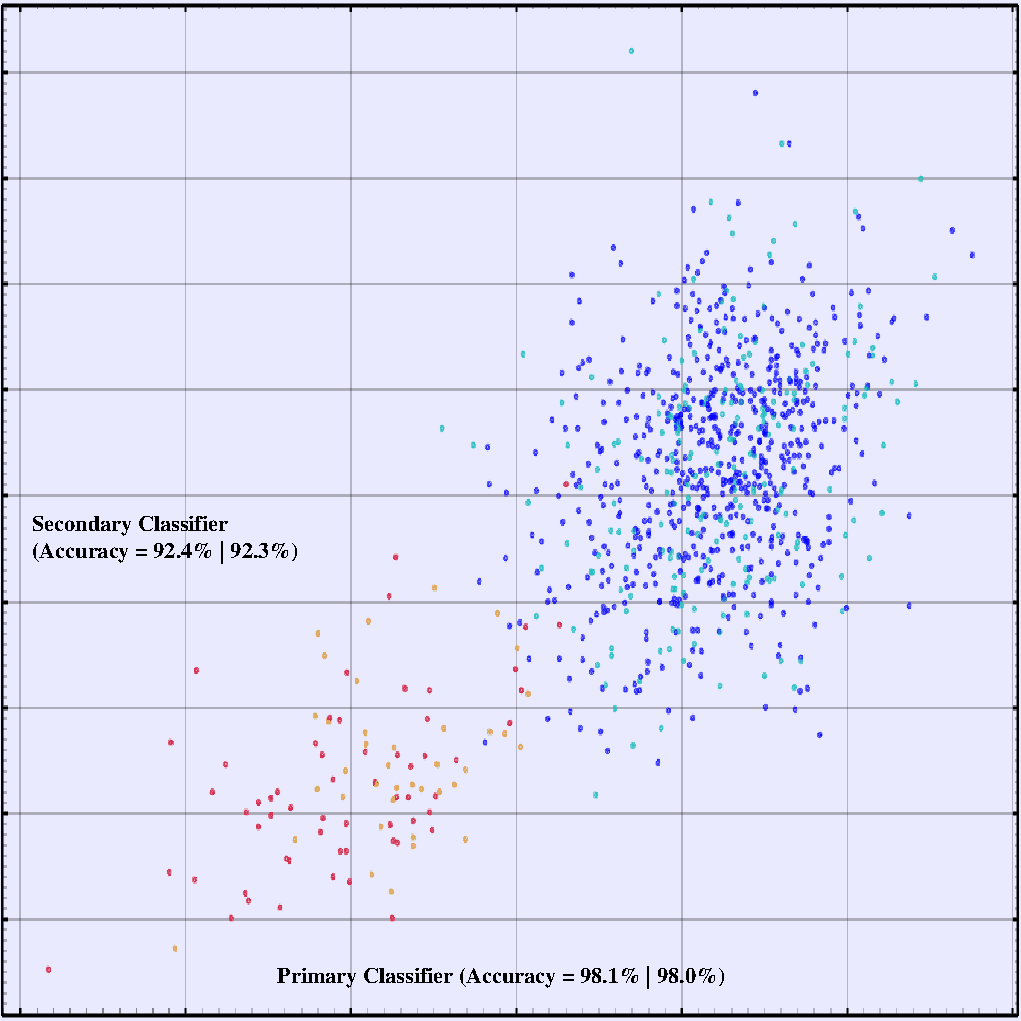 |
| --- |
| Figure B2. **Classification of those with and without history of concussion**. Stepwise linear discriminant analysis (33) was used to classify CamCAN and TEAM-TBI subjects by cohort membership. The classifiers were trained using the baseline resting regional excitability measures. From the total of 169 regional measures for each subject, the analysis selected 12 regions (f-to-remove = 15.996) for the primary classifier: Left (L) fusiform cortex (ctx), L isthmus cingulate ctx, L parahippocampal white matter (wm), L posterior cingulate wm, Right (R) middle temporal wm, R paracentral wm, R precuneus wm, R superior parietal wm, L & R Thalamus, R Cerebellum wm, brainstem.  For the secondary classifier, all regions which were selected in the primary classifier and the corresponding contralateral and ctx/wm regions were eliminated from consideration. From the total of 130 remaining regional measure, the analysis selected 14 regions for the secondary classifier: L lingual ctx, L pars orbitalis ctx, L entorhinal wm, R inferior parietal wm, R caudal anterior cingulate ctx & wm, R rostral anterior cingulate ctx & wm, L & R insula ctx, R Accumbens, R Hippocampus, L cingular angular bundle, L uncincate fasciculus.  The weighting of each subject’s resting recording regional measures on the primary (x-axis) and secondary (y-axis) classifiers are shown in the plot. The training set, i.e. the baseline resting results are plotted in blue (CamCAN: *n* = 614) and red (TEAM-TBI: *n* = 63). The corresponding follow-up resting results are plotted in cyan and orange, *n* = 214, 39. The classification accuracy for each group is listed in Table C2. |

### B3. Classification of the concussed with and without symptoms

MEG-derived patterns of regional excitability were tested for their ability to classify each TEAM-TBI subject for each of six symptoms using stepwise linear discriminant analysis (33). After examination of all neuroimaging and test battery findings, the symptoms were identified by consensus at adjudication by the clinical members of the TEAM-TBI research team.

The symptom assessments for both baseline (*n* = 60) and follow-up (*n* = 36) were pooled for this analysis. The statistical power with *n* of 96 was not sufficient to (a) use disjoint training and test data sets for the classification or (b) to provide confidence that the set of regions whose measures were selected for inclusion in the classifier is complete. All 96 subjects were used to train the classifiers and jackknifed classification was used to test the classifiers. It’s noteworthy that the classifiers for those with and without concussion described above in supplement C2 classified almost all of the follow-up subject correctly but these symptom-specific classifiers distinguish between those with and without symptoms with high accuracy. This elevates the potential importance of expanding the pool of chronic concussed subjects to provide the statistical power to delineate the set of regions which contribute to these classifiers.

| Symptom | correct / *n* | % correct | p-value |
| --- | --- | --- | --- |
| Cognitive | 87 / 96 | 90.6% | p < 10^-16^ |
| Headache | 96 / 96 | 100% | p < 10^-28^ |
| Oculomotor | 91 / 96 | 94.8% | p < 10^-21^ |
| Psychological Health | 90 / 96 | 93.8% | p < 10^-19^ |
| Sleep | 86 / 96 | 89.6% | p < 10^-15^ |
| Vestibular | 90 / 96 | 93.8% | p < 10^-19^ |
| Table B3. **Classification of the concussed with and without symptoms.** 169 regional brain measures were trained as linear classifiers by group determined by adjudicated opinions of clinical symptoms. Jackknifed classification accuracies are shown for all baseline (n = 60) and follow-up (n = 36) TEAM-TBI recordings taken together, i.e. total n = 96. The baseline symptom trajectories were coded 0/1. For follow-up, the code was set to 0 if the subject had improved (ocular) or fully re-covered (all others). Regional excitability measures enable classification of each symptom with high accuracy in patients with multiple chronic symptoms. | | | |

## Supplement C: Theories of concussion

Many hypothesized pathophysiological mechanisms have been proposed, a few as theories but most as experimental hypotheses directed at a single mechanism. The proposed mechanisms fall into three categories: (a) combined biochemical/biomechanical/physiological, (b) biochemical, (c) and biomechanical. With the exception of the reticular hypothesis, each has, as foundational assumptions, (a) that the mechanical impulse of the blow to the head is the initiating cause for any symptoms and (b) that the patient has suffered a brain injury. Hence none of them is applicable to patients without resultant brain injury, a group which may constitute as much as 75% of all mild TBI, i.e. as much as 60% of all head trauma (34).

The five theories are, (1) the vascular hypothesis, (2) the reticular hypothesis, (3) the centripetal hypothesis, (4) the pontine cholinergic hypothesis, and (5) the convulsive hypothesis. All five are primarily aimed at accounting for loss of consciousness although (1), (3), and (5) also account for short-duration amnesia. Detailed reviews of all five are found in (35) .

### Combined biochemical/physiological theories.

The **vascular hypothesis** asserts that loss of consciousness is due to brief cerebral ischemia, perhaps due to vasospasm or momentary brain compression with resultant stasis of blood movement (36). The resultant cerebral ischemia may also account for retrograde amnesia (37). The greatest difficulty with this theory is that the rapid onset of loss of consciousness does not comport with numerous reports of preserved function for many seconds or longer in brain tissue with imposed static blood flow (35). Regardless of the theory’s problems, there is no question that ischemia has a significant role to play in many head injuries, most notably when a hemorrhage, depressed skull fracture, or infarction occurs.

The **reticular hypothesis** asserts that loss of consciousness is due to depression of the electrical activity of the ascending reticular activating system (ARAS) (38). The ARAS is composed of several neural circuits which include the thalamus and cerebral cortex and which originates in the a collection of brainstem nuclei (39) including the locus coeruleus (40), the dorsal and median raphe, and the pedunculopontine and parabrachial nuclei (41). A primary difficulty with this theory is that it is not possible to test in humans with direct electrophysiological recordings. An additional problem is the absence of an accepted mechanism by which a concussive insult can depress reticular function. If verified, sensory induced brainstem crosstalk as proposed in this paper would solve this latter problem. Furthermore, the coma-specific pontine nucleus we mention in the context of loss of consciousness may be one of the originating ARAS brainstem nuclei.

The **centripetal hypothesis** emphasizes the importance of rotational stresses and strains attendant with a blow to the head (42). Is highlights the effects on the cerebral cortex, since that is where the stresses and strains of rotation are maximized. Its applicability is limited because the effects of those mechanical forces on the tissue, must cause injury to produce symptoms. Nevertheless, the ascendent importance of rotational force on white matter injury is an established fact (43).

Acetylcholine (ACh) is a neurotransmitter found in brainstem nuclei which depress/inhibit other neural structures. Release of ACh from injured brainstem neurons is central to the **pontine cholinergic system hypothesis**. This hypothesis is based on the observation that abnormally high concentrations of ACh are present in cerebrospinal fluid in concussed experimental animals and in humans following craniocerebral injury (35).

A substantial problem with the theory is that it presumes that brainstem neurons containing ACh are excited by concussion. But that is quite a different thing than the known fact that ACh is released from broken axons caused by brainstem injury. Furthermore, 40 years of work has failed to establish that excitation of the ACh pontine nuclei consistently effects level of consciousness. Finally, this theory is entirely dependent on the presence of brainstem injury, which confines its applicability to the small percentage of head injuries with the greatest severity.

The **convulsive theory** begins with the fact that marked similarities exist between the acute symptoms of concussion, generalized seizure, and electroconvulsive therapy (35), viz. immediate loss of consciousness with flaccidity followed, in some cases by disorientation, restlessness, lethargy, short term amnesia. The primary problems with this theory are twofold. (1) The mechanism by which mechanical stress is converted into epileptiform neural activity is speculative and the research into mechanism is heavily dependent on concussion which injures the tissue in animals. (2) Only acute symptoms are accounted for by the theory. Post-traumatic seizures do occur, however, in a significant number of patients, although the incidence is considerably lower for low severity TBI (44-46).

### Biochemical mechanisms

There are presently two active and rapidly advancing investigational areas in the biochemistry of concussion. The first is the identification of biochemical mechanisms of degenerative brain disease that may be common to repeated concussion resulting in chronic traumatic encephalopathy (CTE), Parkinson’s disease, and Alzheimer’s disease. The second is the identification of serum biomarkers of brain injury.

The putative mechanism(s) by which repeated concussions lead to CTE is beyond the scope of this paper. This, however, is an important line of research, not just for concussed patients at risk for CTE, but for what may be learned which could provide breakthroughs in other degenerative brain diseases.

The search for reliable blood born biomarkers of brain injury has led to federal approval for evaluation of patients in the 24 hours following concussion (47). The Abbott test is an assay for two proteins which normally reside only in the brain, GFAP and UCH-L1. These molecules are only detectable in the blood when the blood-brain barrier is broken, i.e. when there is a brain injury. The test has been approved because studies from many groups have shown that the correlation is consistently near 90% or higher between a positive biomarker finding and a positive computed tomography (CT) neuroimaging study (48,49).

The ability to identify brain injury using neuroimaging has progressively improved, yet 70-75% of all patients with mild TBI still have negative neuroimaging findings (50). Perhaps that 25-30% diagnostic rate for brain injury has plateaued, not because neuroimaging modalities lack sufficient sensitivity, but because those mild TBI patients with negative neuroimaging findings in fact have no brain injury.

Of course, it is possible that CT and the UCH-11/GFAP assay coincidently have nearly the same sensitivity to the same brain injuries. Even so, it make sense to explore theories which do not rely on an undetectable phenomenon in well over half the patients, viz. brain injury. This is one of the motivations for the development of this theory of sensory-induced neurophysiological mechanisms of concussion.

### Biomechanical mechanisms

Biomechanics has dominated concussion research for more than a century. The understanding of the intracranial pathology invariably is based on an injury model. Examples include elevated intracranial pressure due to cortical bruising (51), tissue damage due to propagation of the concussion pressure wave through the noncompressible fluid within the rigid confines of the skull (36), ischemia due to swelling (38), local breakage due to the distortion caused by rotational forces (42), and many more. The governing concept which underlies all of these is that the harder the blow to the head, the more severe will be the injury.

The introduction of helmets and other protective devices for military personnel, cyclists, motorcyclists, hockey players, etc. is an effort to reduce the impact of concussion and mitigate its effects. The ideal protective gear would reduce the impact below the threshold for brain injury. And certainly, there is such a threshold. The magnitude of the helmet research effort (52,53) reflects a long term commitment to perfect and validate these designs. Relevant to this discussion, the assertion which underlies the sensory driven neurophysiological theory presented in this paper is that even in the impact regime below which no brain injury occurs, significant symptomatic sequelae of concussion can and do occur.

## References

1. Watson. Ocular Anatomy and Physiology Relevant to Anaesthesia. 12, 2019, Anaesthesia and Intensive Care Medicine, Vol. 20, pp. 710-715.

2. Arnold, RW. The Oculocardiac Reflex: A Review. 2021, Clinical Ophthalmology, Vol. 15, pp. 2693-2725.

3. André Kahn, Jalil Riazi, Denise Blum; Oculocardiac Reflex in Near Miss for Sudden Infant Death Syndrome Infants. 1983, Pediatrics, Vol. 71, pp. 49-52.

4. Henry, L. C. , Elbin, R. , Collins, M. W. , Marchetti, G. & Kontos, AP. Examining Recovery Trajectories After Sport-Related Concussion With a Multimodal Clinical Assessment Approach., 2016, Neurosurgery, Vol. 78, pp. 232-241.

5. AL Santo, ML Race, EF Tee. Near Point of Convergence Deficits and Treatment Following Concussion: A Systematic Review. 2020, J Sport Rehab, Vol. 28, pp. 1179-1193.

6. AP Kontos, SR Eagle, A Mucha, V Kochick, J Reichard, C Moldolvan, CL Holland, NA Blaney, MW Collins. A Randomized Controlled Trial of Precision Vestibular Rehabilitation in Adolescents Following Concussion: Preliminary Findings. 2021, J Pediatrics, Vol. 239, pp. 193-199.

7. Tyler CW, Likova LT, Mineff KN and Nicholas SC. Deficits in the activation of human oculomotor nuclei in chronic traumatic brain injury. 2015, Front Neurology, Vol. 6.

8. Bronstein, A., Patel, M. & Arshad, Q. A brief review of the clinical anatomy of the vestibular-ocular connections—how much do we know. 2015, Eye, Vol. 29, pp. 163-170.

9. Sanchez, K. and Rowe, F.J. Role of neural integrators in oculomotor systems: a systematic narrative literature review. 2018, Acta Ophthalmol, Vol. 96, pp. e111-e118.

10. Liston, Dorion B., and Leland S. Stone. Oculometric Assessment of Dynamic Visual Processing. 14, 2014, J Vision, Vol. 14, p. 12.

11. Ivanchenko D, Rifai K, Hafed ZM, Schaeffel F. A low-cost, high-performance video-based binocular eye tracker for psychophysical research. 2021, J eye Mov Res, Vol. 14.

12. Markus Kofler, Mark Hallett, Gian Domenico Iannetti, Viviana Versace, Jens Ellrich, Maria J. Téllez, Josep Valls-Solé. The blink reflex and its modulation – Part 1: Physiological mechanisms. 2024, Clin Neurophys, Vol. 160, pp. 130-152.

13. Garner, Dena. Pilot Study to Assess Changes in the Blink Reflex Across Age. suppl 1, Neurology, Vol. 98.

14. Garner, D. P., Goodwin, J. S., Tsai, N. T., Kothera, R. T., Semler, M. E., Wolf, B. J., & Jin, Z. Blink reflex parameters in baseline, active, and head-impact Division I athletes. 2018, Cogent Engineering, Vol. 5.

15. Garner, D. P., Sparks, P. D., Chizuk, H. M., & N. Haider, M. Effect of exertion on blink reflex parameters in Division I football athletes. 2023, Cogent Engineering, Vol. 10.

16. J E Dugan, J Jo, C C Long, K L Williams, S L Zuckerman, D P Terry. Changes in the Blink Reflex after a Sport-Related Concussion: Test–Retest Reliability of a Blink Reflexometer. 2024 : s.n., Archives of Clinical Neuropsychology,.

17. AM Yengo-Kahn, DP Garner, N Lessing, J Blough, SL Zuckerman, K Gifford. Normative blink reflex data for the EyeStat Device in student athlete. 2022, Cogent Engg, Vol. 9.

18. Russell E. Banks, Deryk S. Beal & Eric J. Hunter. Sports Related Concussion Impacts Speech Rate and Muscle Physiology, Brain Injury. 2021, Vol. 35, pp. 1275-1283.

19. Nina Kraus1, ElaineC.Thompson, Jennifer Krizman, KatherineCook, TravisWhiteSchwoch & Cynthia R. LaBella. Auditory biological marker of concussion in children. 2016, Nature Scientific Reports.

20. Jaehong Park , Seonmi Choi, Jun Takatoh, Shengli Zhao, Andrew Harrahill, Bao-Xia Han, and Fan Wang. Brainstem Control of Vocalization and Its Coordination with Respiration. 2024, Science, Vol. 383, p. 8081.

21. G.M. Schulz, M. Varga, K. Jeffires, C.L. Ludlow, A.R. Braun. Functional Neuroanatomy of Human Vocalization: An H215O PET Study. 2005, Cerebral Cortex, Vol. 15, pp. 1835-1847.

22. Veerakumar, A., Head, J.P. & Krasnow, M.A. A brainstem circuit for phonation and volume control in mice. 2023, Nat Neurosci, Vol. 26, pp. 2122-2130.

23. C Poellabauer, N Yadav, L Daudet, SL Schneider, C Busso, PJ Flynn. Challenges in Concussion Detection Using Vocal Acoustic Biomarkers. 2015, IEEE Access, Vol. 3, pp. 1143-1160.

24. Cai, H. and Ternström, S. Mapping Phonation Types by Clustering of Multiple Metrics. 2022, Appl Sci, Vol. 12.

25. A. Jean. Brain stem control of swallowing: neuronal network and cellular mechanisms. 2001, Physiol Rev, Vol. 2, pp. 929-969.

26. Hiroaki Hashimoto, Masayuki Hirata, Kazutaka Takahashi, Seiji Kameda, Yuri Katsuta, Fumiaki Yoshida, Noriaki Hattori, Takufumi Yanagisawa, Jason Palmer, Satoru Oshino, Toshiki Yoshimine & Haruhiko Kishima. Non-invasive quantification of human swallowing using a simple motion tracking system. 2018, Sci Rep, Vol. 8.

27. Smith SS, Jahn KN, Sugai JA, Hancock KE, Polley DB. The human pupil and face encode sound affect and provide objective signatures of tinnitus and auditory hypersensitivity disorders. January 2024, bioRxiv [Preprint.

28. Tripathi SC, Garg R. Consistent movement of viewers’ facial keypoints while watching emotionally evocative videos. 2024, PLoS ONE, Vol. 19.

29. D Krieger, P Shepard, R Soose, A Puccio, S Beers, W Schneider, AP Kontos, MW Collins, DO Okonkwo. MEG-Derived Symptom-Sensitive Biomarkers with Long-Term Test-Retest Reliability. 2022, Diagnostics, Vol. 12.

30. Fischl, B., Sereno, M.I. and Dale, A. Cortical Surface-Based Analysis: II: Inflation, Flattening, and a Surface-Based Coordinate System. 1999, NeuroImage, Vol. 9, pp. 195-207.

31. Reuter, M.; Schmansky, N.J.; Rosas, H.D.; Fischl, B. Within-subject template estimation for unbiased longitudinal image analysis. 2012, NeuroImage, Vol. 61, pp. 1402-1418.

32. Yendiki, A.; Panneck, P.; Srinivasan, P.; Stevens, A.; Zöllei, L.; Augustinack, J.; Wang, R.; Salat, D.; Ehrlich, S.; Behrens. Automated probabilistic reconstruction of white-matter pathways in health and disease using an atlas of the underlying anatomy. 2011, Front Neuroinform, Vol. 5.

33. WJ Dixon. Chapter on BMDP7M. [book auth.] BMDP Statistical Software Manual. Berkeley : University of California Press, 1990.

34. Dewan MC, Rattani A, Gupta S, Baticulon RE, Hung YC, Punchak M, Agrawal A, Adeleye AO, Shrime MG, Rubiano AM, Rosenfeld JV, Park KB. Estimating the global incidence of traumatic brain injury. 2018, J Neurosurgery, Vol. 130, pp. 1080-1097.

35. NA Shaw. The Neurophysiology of Concussion. 2002, Progress Neurobio, Vol. 67, pp. 281-344.

36. WR Russell. Experimental cerebral concussion. 1940, J Physiology, Vol. 99, p. 153.

37. Ommaya, A. K., Rockoff, S. D., Baldwin, M., & Friauf, W. S. Experimental Concussion: A First Report. 1964, J Neurosurgery, Vol. 21, pp. 249-265.

38. Foltz, E. L., Jenkner, F. L., & Ward, A. A., Jr. Experimental Cerebral Concussion. 1953, J Neurosurgery, Vol. 10, pp. 342-352.

39. Yeo SS, Chang PH and Jang SH. The ascending reticular activating system from pontine reticular formation to the thalamus in the human brain. 2013, Front Human Neurosci, Vol. 7.

40. Aston-Jones G, Chen S, Zhu Y, Oshinsky ML. A neural circuit for circadian regulation of arousal. 2001, Nat Neurosci, Vol. 4, pp. 732-738.

41. Josef Parvizi, Antonio R. Damasio. Neuroanatomical correlates of brainstem coma. 2003, Brain, Vol. 126, pp. 1524-1536.

42. Ommaya AK, Gennarelli TA. Cerebral concussion and traumatic unconsciousness. Correlation of experimental and clinical observations of blunt head injuries. 1974, Brain, Vol. 97, pp. 633-654.

43. Patel H, Polam S, Joseph R. Overview of Treatment Options for Mild Traumatic Brain Injury: A Literature Review. 2024, Cureus, Vol. 16.

44. Frey, LC. Epidemiology of posttraumatic epilepsy: a critical review. 2003, Epilepsia, Vol. 44, pp. 11-17.

45. Anwer F, Oliveri F, Kakargias F, Panday P, Arcia Franchini AP, Iskander B, Hamid P. Post-Traumatic Seizures: A Deep-Dive Into Pathogenesis. 2021, Cureus, Vol. 13.

46. Kazis, D.; Chatzikonstantinou, S.; Ciobica, A.; Kamal, F.Z.; Burlui, V.; Calin, G.; Mavroudis, I. Epidemiology, Risk Factors, and Biomarkers of Post-Traumatic Epilepsy: A Comprehensive Overview.2024, Biomedicines, Vol. 12.

47. Geoffrey Manley, MD, PhD, PI for Track-TBI Network; Beth McQuiston, M.D., medical director in Abbott's diagnostics. Abbott Receives FDA Clearance for Whole Blood Rapid Test to Help with Assessment of Concussion at the Patient's Bedside. Federal Drug Administration. Abbott, 1Apr2024. Press Release.,.

48. Linda Papa, Jay G. Ladde, John F. O’Brien, Josef G. Thundiyil, James Tesar, Stephen Leech, David D. Cassidy, Jesus Roa, Christopher Hunter, Susan Miller, Sara Baker, Gary A. Parrish, M. Evaluation of Glial and Neuronal Blood Biomarkers Compared With Clinical Decision Rules in Assessing the Need for Computed Tomography in Patients With Mild Traumatic Brain Injury. 2022, JAMA Network Open, Vol. 5.

49. D Janigro, S Mondello, JP Post, J Unden. What You Always Wanted to Know and Never Dared to Ask. 2022, Frontiers in Neurology, Vol. 13.

50. Mayer AR, Cohen DM, Wertz CJ, Dodd AB, Shoemaker J, Pluto C, Zumberge NA, Park G, Bangert BA, Lin C, Minich NM, Bacevice AM, Bigler ED, Campbell RA, Hanlon FM, Meier TB, Oglesbee SJ, Phillips JP, Pottenger A, Shaff NA, Taylor HG, Yeo RA, Arbogast KB. Radiologic common data elements rates in pediatric mild traumatic brain injury. 2020, Neurology, Vol. 94, pp. e241-e253.

51. Trotter, W. On Certain Minor Injuries Of The Brain. Being The Annual Oration, Medical Society Of London. 1924, Brit Med J, Vol. 1, pp. 816-819.

52. Rowson, B., Duma, S.M. A Review of On-Field Investigations into the Biomechanics of Concussion in Football and Translation to Head Injury Mitigation Strategies. 2020, Vol. 48, pp. 2734-2750.

53. Tierney, G. Concussion biomechanics, head acceleration exposure and brain injury criteria in sport: a review. 2021, Sports Biomechanics, pp. 1-29.
